# Changes in Virtual and In-Person Health Care Utilization in a Large Health System During the COVID-19 Pandemic

**DOI:** 10.1001/jamanetworkopen.2021.29973

**Published:** 2021-10-27

**Authors:** Kori S. Zachrison, Zhiyu Yan, Lee H. Schwamm

**Affiliations:** 1 Department of Emergency Medicine, Massachusetts General Hospital, Boston; 2 Harvard Medical School, Boston, Massachusetts; 3 Department of Neurology, Boston, Massachusetts

## Abstract

This cross-sectional study evaluates how the growth of virtual care has impacted health care utilization in an integrated delivery network.

## Introduction

Virtual care has quickly become an integral component of care delivery.^1^ However, some have expressed legitimate concern that this may increase health care spending by leading to churn and increase total medical expense.^2,3^ As the pandemic and associated regulatory relief led to more permissive use of virtual care, we sought to evaluate the association between the growth of virtual care and health care utilization in an integrated delivery network.

## Methods

This cross-sectional study was approved by the institutional review board at Massachusetts General Hospital and follows the Strengthening the Reporting of Observational Studies in Epidemiology (STROBE) reporting guideline. Informed consent was not required because this was a deidentified analysis of existing medical records data.

We used data from our large New England health care system (ie, 12 hospitals and their outpatient practice sites) to identify all ambulatory visits between October 1, 2019, to April 30, 2021. Encounters were virtual, in-person at home, and in-person onsite. We also analyzed the subgroup of behavioral health care professional visits to identify any unique behavioral health visit trends.

Race and ethnicity data were included to characterize the demographics of our population served. Race and ethnicity data were extracted from the electronic health record. We constructed a composite, mutually exclusive race and ethnicity variable based on individual race and ethnicity fields in the electronic health record because some patients had reported Hispanic ethnicity but had not reported race. Categories included Hispanic, missing, non-Hispanic Asian, non-Hispanic Black, non-Hispanic White, and other. The category *other* included all individuals of a race or ethnicity that was not listed, such as Alaskan Native or American Indian.

We used a 2-sided Welch *t* test to compare overall mean monthly visit volume prepandemic (October 1, 2019, to February 1, 2020) vs current (June 1, 2020, to April 30, 2021) and stratified by encounter type (ie, virtual, in-person at home, and in-person onsite). We excluded March to May 2020 as the lower visit volumes during those months were related to the pandemic onset and not reflective of current use trends. Statistical significance was set at *P* < .005, and analysis was performed with R version 4.0.2 (R Project for Statistical Computing). We secondarily examined patient-level trends (eAppendix in the Supplement).

## Results

After excluding 29 visits with unclear modality (ie, virtual vs in-person), we identified 10 559 857 ambulatory visits during the study period by 1 530 772 patients (median age, 58 years; IQR, 37-71 years; 918 463 females [60%]; 612 309 males [40%]). The sample’s racial and ethnic demographics included 61 231 (4%) Asian patients, 122 462 (8%) Hispanic patients, 91 846 (6%) non-Hispanic Black patients, 1 178 693 (77%) non-Hispanic White patients, and 76 540 (5%) patients of an unknown or other race and ethnicity. Of these patients, 811 309 (53%) had only in-person; 627 617 (41%) had in-person and virtual; and 91 846 (6%) had only virtual visits. Among patients with both in-person and virtual visits, most conducted fewer than half of the visits virtually (median, 33%; IQR, 20%-50%). Of all encounters, 23.8% were virtual, 8.2% were in-person at home, and 68.0% were in-person onsite.

At the onset of the pandemic, the number of in-person visits fell dramatically while virtual visits increased overall and among behavioral health visits (Figure). We found no significant change in overall ambulatory visit volume, and visit patterns were similar at the patient level (Table). Among behavioral health visits from October 1, 2019, to April 30, 2021, we found a small increase in the total visit volume, which was driven by increased virtual visits (mean monthly visit volume increased from 29 609 to 36 901; *P* < .002; with 94% of monthly visits being virtual after May 2020) (Table).

**Figure.**
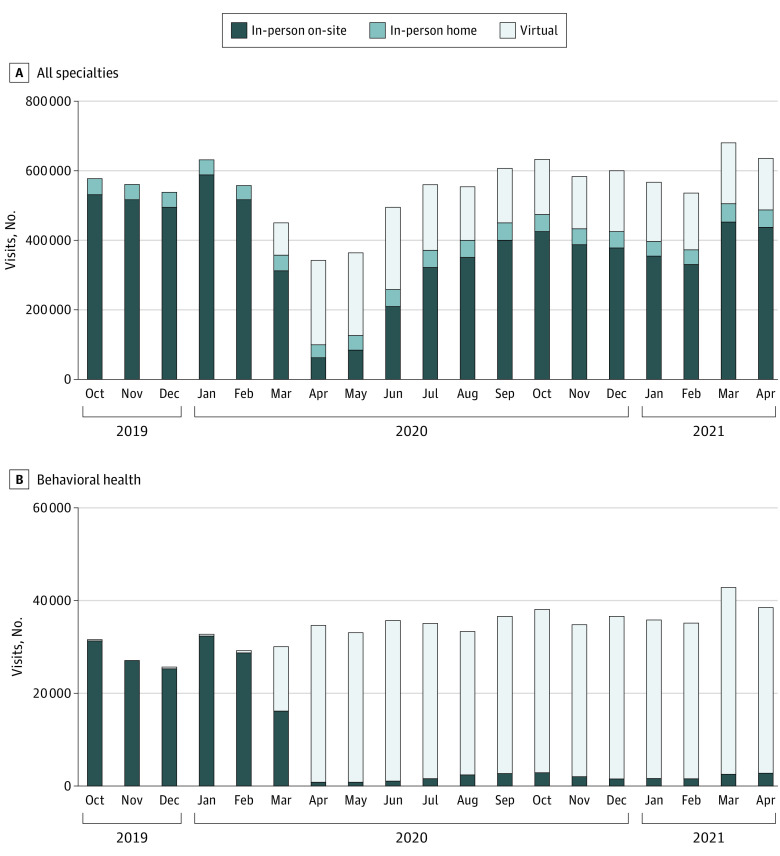
Monthly Total Ambulatory Visit Volume by Visit Type (In-Person Plus Virtual) from October 1, 2019, to April 30, 2021

**Table.** Comparison of Mean Monthly Visit Volumes Prepandemic vs Current

Visits	Mean monthly visit volume		*P* value for difference^a^
	October 2019-February 2020	June 2020-April 2021	
All visits, No. (% of all visits)			
Total, No.	578 084	590 613	.59
Virtual	1488 (0.3)	174 579 (29.6)	<.001
In-person home	46 439 (8.0)	46 796 (7.9)	.81
In-person on-site	530 156 (91.7)	369 239 (62.5)	<.001
Mean No. of visits per patient per month	1.7	1.8	.08
Behavioral health visits (% of all behavioral health visits)			
Total, No.	29 609	36 901	.002
Virtual	552 (1.9)	34 673 (94.0)	<.001
In-person	29 057 (98.1)	2229 (6.0)	<.001

^a^ Statistical significance was set at *P* < .05.

## Discussion

Our results suggest that the transition to incorporate virtual care into our ambulatory care offerings was not associated with increased overall visit volumes. We believe this suggests that, to date, virtual care has been substitutive, rather than additive, within our system and provided a vital avenue for care delivery. We are unable to quantify the true amount of care needed by patients during this time, but it is likely that virtual care offerings lowered barriers in access to care during the period of limited in-person gatherings. This may be particularly true for behavioral health, with a near-complete shift from in-person to virtual visits. Given the impact of the pandemic on mental health^4^ and known preexisting challenges in access to behavioral health access in general,^5^ the increased volume of behavioral health visits in our system over the study period may be merited and high value overall.

This study had limitations. These findings are limited to a single regional health system and the effects of the pandemic are ongoing, and we did not examine cost or other use associated with ambulatory visits (eg, testing). However, our finding of stable ambulatory visit volumes with the introduction of virtual care offerings may provide early reassurance to policymakers and payors. Further work is warranted to better understand changes in use, total medical expense, clinical outcomes, and the relative value contributed by these visits to patients and the health system.

## Notes

Supplement.
eMethods.
