# Supplementary material for: Changes in Virtual and In-Person Health Care Utilization in a Large Health System During the COVID-19 Pandemic
Source: JAMA Netw Open. 2021 Oct 27;4(10):e2129973. doi: 10.1001/jamanetworkopen.2021.29973 (PMC8552053; doi:10.1001/jamanetworkopen.2021.29973)
Supplement: Supplement. — eMethods. [file jamanetwopen-e2129973-s001.pdf]

Supplemental Online Content

Zachrison KS, Yan Z, Schwamm LH. Changes in virtual and in-person health care utilization in a large health system during the COVID-19 pandemic. *JAMA Netw Open*. 2021;4(10):e2129973. doi:10.1001/jamanetworkopen.2021.29973

eMethods.

This supplemental material has been provided by the authors to give readers additional information about their work.

© 2021 Zachrison KS et al. *JAMA Network Open.*

eMethods

We were also interested in trends at the patient-level. Because most patients in the data did not have encounters every month, we randomly divided the entire sample into cohorts of 200 patients each and assigned them to 7,654 unique groups (with the last group having 172 patients). We then plotted the group-level transition over time by generating weekly visit volumes for each of the 7,654 groups and examining these volumes overall and then stratified by virtual versus in-person.

© 2021 Zachrison KS et al. *JAMA Network Open.*
